# A viral race for primacy: co-infection of a natural pair of low and highly pathogenic H7N7 avian influenza viruses in chickens and embryonated chicken eggs

**DOI:** 10.1038/s41426-018-0204-0

**Published:** 2018-12-05

**Authors:** Annika Graaf, Reiner Ulrich, Pavlo Maksimov, David Scheibner, Susanne Koethe, Elsayed M. Abdelwhab, Thomas C. Mettenleiter, Martin Beer, Timm Harder

**Affiliations:** 1 Institute of Diagnostic Virology, Südufer 10, 17493 Greifswald, Germany; 2 Department of Experimental Animal Facilities and Biorisk Management, Südufer 10, 17493 Greifswald, Germany; 3 Institute of Epidemiology, Südufer 10, 17493 Greifswald, Germany; 4 Institute of Molecular Virology and Cell Biology, Südufer 10, 17493 Greifswald, Germany

## Abstract

Highly pathogenic avian influenza virus (HPAIV) infection in poultry caused devastating mortality and economic losses. HPAIV of subtypes H5 and H7 emerge from precursor viruses of low pathogenicity (LP) by spontaneous mutation associated with a shift in the susceptibility of the endoproteolytic cleavage site of the viral hemagglutinin protein from trypsin- to furin-like proteases. A recently described natural pair of LP/HP H7N7 viruses derived from two spatio-temporally linked outbreaks in layer chickens was used to study how a minority of mutated HP virions after de novo generation in a single host might gain primacy. Co-infection experiments in embryonated eggs and in chickens were conducted to investigate amplification, spread and transmissionof HPAIV within a poultry population that experiences concurrent infection by an antigenically identical LP precursor virus. Simultaneous LPAIV co-infection (inoculum dose of 10^6^ egg-infectious dose 50% endpoint (EID_50_)/0.5 mL) withincreasing titers of HPAIV from 10^1^ to 10^5.7^ EID_50_/0.5 mL) had a significant impeding impact on HP H7 replication, viral excretion kinetics, clinical signs and histopathological lesions (in vivo) and on embryo mortality (in ovo). LP/HP co-infected chickens required a hundredfold higher virus dose (HPAIV inoculum of 10^5^ EID_50_) compared to HPAIV mono-infection (HPAIV inoculum of 10^3^ EID_50_) to develop overt clinical signs, mortality and virus spread to uninfected sentinels. Escape and spread of HP phenotypes after de novo generation in an index host may therefore be highly precarious due to significant competition with co-circulating LP precursor virus.

## Introduction

Avian influenza A viruses (AIV) are classified into subtypes based on antigenic properties of their hemagglutinin (HA) and neuraminidase (NA) surface glycoproteins^1^. A further classification into phenotypes of low and high pathogenicity (LP/HP) refers to their virulence in chickens. In the subtypes H5 and H7, the HP phenotype correlates with presence of multiple basic amino acids at the HA endoproteolytic cleavage site (HACS) rendering it accessible to subtilisin-like proteases that are ubiquitous in avian host tissues^2–4^.The vast majority of AIV circulating in their natural host reservoir of aquatic wild birds is identified as LP, encoding monobasic, i.e., trypsin-sensitive HACSs, and causing only minor, if any, clinical signs in avian hosts, including poultry^5^. LP viruses of the subtypes H5 and H7 have the ability, under natural conditions, to spontaneously mutate to the HP phenotype which is associated with conversion of the HACS from a trypsin-sensitive/monobasic to a subtilisin-sensitive/polybasic configuration^6^. The concept of HPAIV emergence from LP progenitors is supported by phylogenetic analyses of H5 and H7 AIV strains, which revealed the evolution of geospatially defined lineages within LPAIV, but not HPAIV phenotypes^7,8^. So far, only subtypes H5 and H7 AI viruses have been observed to acquire HP mutations under natural circumstances, and infection of poultry with these viruses are therefore considered to be notifiable regardless of their particular pathogenicity^9,10^. Control of notifiable LPAIV of subtypes H5 and H7 in poultry aims at preventing spontaneous mutation to and spread of HPAI variants associated with vast economic losses in poultry production. Some LPAIV such as the H7N9 strain in China merit control also because of their unique zoonotic propensity^11^.

Repeated, but epidemiologically unrelated, emergence of HPAI H7 viruses based on de novo generation from distinct LPAIV precursors was at the basis of major outbreaks in Italy 1999–2000 (H7N1; ref.^12^) and 2013 (H7N7; ref.^13^), in the Netherlands in 2003 (H7N7; refs.^14,15^), in Canada in 2004 (H7N3; ref.^16^), in the United States of America in 2016 (H7N8; ref. ^17^) and 2017 (H7N9; ref.^18^), in the UK in 200819,20 and Germany in 2015 (H7N7; ref.^19^). H7 HPAIV detection in isolated, sporadic outbreaks was reported from the UK in 2015 (H7N7; ref.^20^), from Canada in 2007 (H7N3; ref.^21^) and from Spain in 2009/2010 (H7N7; ref.^22^).

Virological identification of a “matching pair” of an LPAIV progenitor and its HPAIV descendant in the field is very rare^23–25^. A recent example of such a virus pair was reported from Germany where transmission of a precursor H7N7 LPAIV from chicken layer farm A to neighboring layer farm B and mutation to the HP phenotype on farm B was confirmed^19^.Determining the drivers of emergence of HPAIV is crucial for a better understanding why and when certain LP strains pose a risk of becoming HP. There is insufficient knowledge why, in nature, the HP phenotype emerges only in H5 and H7 subtypes and how the two AIV pathotypes interact when simultaneously infecting poultry. We conducted in vivo and in ovo co-infection experiments using a naturally occurring matching LP/HP H7N7 virus pair to further understand the processes of the initial emergence and escape of HPAIV. Our experiments attempted to mimic the situation of the de novo emergence and spread of an HPAIV infection in a chicken population in which LPAIV is circulating.

## Results

In order to mimic the status nascendi,when an HP phenotype variant emerges by spontaneous mutation from an LP precursor virus in an avian host that is infected by the LP phenotype, groups of ten 6-week-old specific pathogen free (SPF) chickens or five SPF embryonated chicken eggs (ECEs) of either 10-days or 14-days of incubation, respectively, were co-inoculated with an HP/LP virus mixture containing increasing titers of HPAIV H7N7 AR1385from 10^1^ to 10^5.7^ EID_50_/animal (groups C1-C5.7, C = co-infected, number = log_10_ virus titer) in a constant background titer (10^6^ EID_50_ /animal) of the LPAIV H7N7 AR915 precursor. As a comparison, similar inoculation of chickens and eggs were carried out with corresponding HPAIV doses without concomitantLPAIV infection (HP mono (M)-infected groups M1-M6). In addition, we also included a group which solely received the LPAIV precursor (group B).

### In vivo experiment

#### Clinical score and survival rate of chickens reveal interference of LP with low titer HP H7N7 infection

**Fig. 1:**
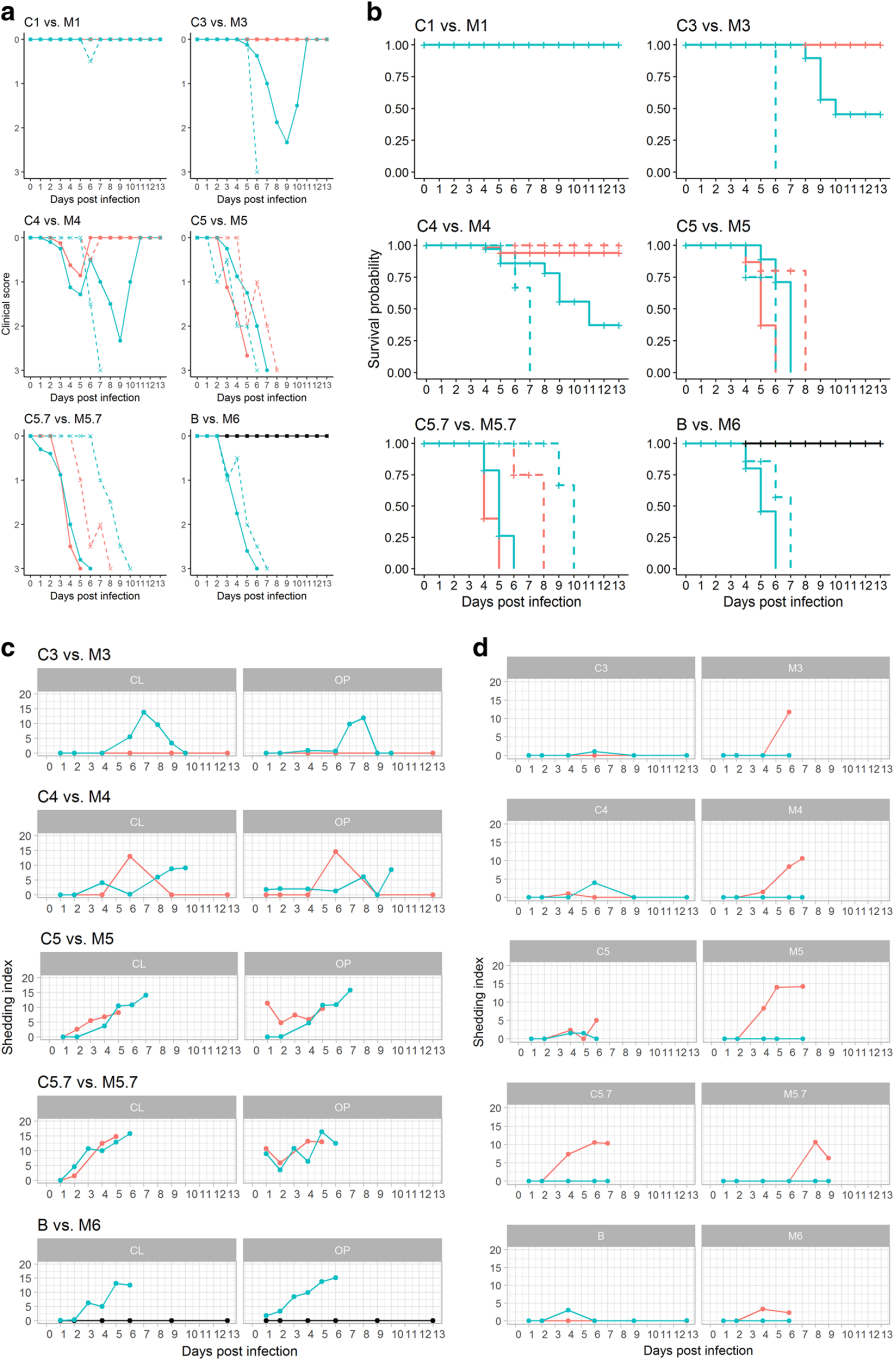
**a**-**b**: Clinical scores (**a**) and survival probability (**b**) of chickens after co-infection with LP/HPAIV H7N7 (groups C1-C5.7, orange lines) compared with titer-matched HP H7N7 mono-infected groups (M1-M5.7, turquoise lines), and comparison between titer-matched LP (B, black line) and HP H7N7 (M6, turquoise line) mono-infected groups. Continuous lines represent inoculated animals; dashed lines depict sentinels. **c–d**: Oropharyngeal (OP) and cloacal (L) shedding of HPAIV H7N7-specific RNA by infected (**c**), and sentinel (**d**) chickens after co-infection with LP/HPAIV H7N7 (groups C1-C5.7, orange lines) compared with titer-matched HP H7N7 mono-infected groups (M1-M5.7, turquoise lines), and comparison between titer-matched LP (B, black line) and HP H7N7 (M6, turquoise line) mono-infected groups. A pathotype-specific RT-qPCR was used to generate Cq values (Graaf, 2017). The shedding index (*Y*-axes) was calculated by computing the mean average of Cq values of all animals sampled at the indicated dpi in a specific group and subtracting this value from 38, the threshold of detection of the HP H7 RT-qPCR. Thus, if all animals were negative for HP H7 RNA, the group scored with a value of zero

Neither morbidity (quantitative measure: clinical score; Fig. 1a) nor mortality (quantitative measure: survival probability, Fig. 1b) was observed in any bird of group B inoculated with 10^6^ EID_50_ of LPAIV H7N7 AR915 as a mono-infection (Fig. 1a, b: B vs. M6, blacklines). Dose-dependent morbidity and mortality was evident in groups that received an inoculum of HPAIV H7N7 AR1385, the HP successor of LPAIV AR915, as a mono-infection (groups M1-M6, Fig. 1a, b, turquoise lines). Animals of group M6 which received the highest HPAIV dose (10^6^ EID_50_/0.5 mL) presented with severe lethargy, anorexia and neurological signs leading to a moribund status as defined in humane termination criteria (see Materials and Methods) of all inoculated birds within 3 to 4 days post infection (dpi) (mean clinical score = 2.1). As expected, highly significant differences between the control groups LP B and HP M6 in survival rates and clinical scores of inoculated animals were observed (each *p* < 0.001) which confirms the validity of the inoculation and scoring system (Fig. 1a, b, B vs. M6, supplemental Table 1a, b).

Average clinical scores of other HPAIV challenged groups decreased with decreasing inoculum titers from 2.0 (in group M5.7), 1.9 (in group M5) and 1.6 (in group M4) to 1.4 (group M3). A delay in mortality but no difference in the terminal severity of clinical signs was observed for the groups M3 and M4 compared to groups M5, M5.7, and M6 (Fig. 1a, b, turquoise lines). However, mortality did not reach 100% in groups M3 and M4 and, thus, survival probability in groups M3 and M4 was significantly higher (each, *p* < 0.001) than in M5-M6. In group M1, in contrast, neither clinical signs nor mortality were observed. In conclusion, as a mono-infection, at least 10^3^ EID50 of the HPAIV AR1385 inoculum per animal were required to induce robust infection, clinical signs and mortality.

Chicken groups C1-C5.7 (Fig. 1a, b, orange lines) had been co-infected with an inoculum containing a constant amount of LP AR915 (10^6^ EID_50_/animal) and varying titers of HP AR1385 matching those of the mono-infected groups. Compared to the HPAIV mono-infected groupsthey showed an attenuated course of disease: HPAIV added to the LPAIV inoculum at doses of 10^1^ EID_50_ (group C1) and also 10^3^ EID_50_ (group C3) did not induce any signs of disease or mortality. Thus, there was a significant difference between group C3 (no morbidity/mortality) vs. M3 (9/10 birds died) regarding survival probability and clinical scores (each *p* < 0.001). An HPAIV co-infection dose of 10^4^ EID_50_ (group C4) infrequently caused very discrete clinical signs in chickens. Spontaneous mortality was induced in two inoculated birds of this group at 4 and 5 dpi that did not show any preceding clinical signs (C4, meanclinical score = 0.6; Fig. 1a, b, orange lines); this reveals a highly significant difference to group M4 (survival probability *p* = 0.002; clinical score *p* = 0.002) (supplementalTable 1a, b).

High morbidity and mortality rates, evidently indistinguishable (survival probability and clinical scores *p* > 0.2) from HPAIV mono-infection, were induced by mixed inocula containing 10^5^ and 10^5.7^ EID_50_ HPAIV in groups C5 and C5.7 (Fig. 1a, b; supplemental Table 1a, b), respectively.

The only significant difference emerged within the survival rates between groups C5 vs. M5 in inoculated animals (*p* << 0.001). In conclusion, when compared to HPAIV mono-infections, an attenuated course of disease was evident when less than 10^5^ EID_50_ of the HPAIV were mixed with an inoculum of 10^6^ EID_50_ of the LPAIV.

#### HP H7N7 virus shedding is impeded by LP co-infection

Oropharyngeal (OP) and cloacal (CL) viral shedding patterns were examined by generic AIV-M-specific and pathotype-specific H7 LP and H7 HP RT-qPCRs (Hoffmann, 2001; Graaf et al., 2017). The total number of positive swabs, the viral loads and the duration of virus shedding varied among the groups (Fig. 1c and supplemental Table 2). The threshold distinguishing positive and negative samples was set at Cq = 40 for the M- and at 38 for both, the H7 LP and HP specific PCRs. The latter PCRs were found to be slightly less sensitive leading to more M-positive versus H7-positive samples (supplemental Table 2). No H7 LPAIV-specific RNA was detected in any of the HPAIV mono-infected groups M1-M6, and likewise, no H7 HPAIV-specific RNA was detected in the LPAIV mono-infected group B, confirming the purity of the virus preparations and effective isolation of animal units.

All virus-inoculated chickens with the exception of group M1 and a single inoculated chicken in group M3 excreted AIV RNA in OP and/or CL swabs (supplemental Table 2). Chickens of the LPAIV mono-infected group B shed virus orally and cloacally starting from 1 and 2 dpi, respectively, and virus excretion in this group peaked at 4 dpi; even at 13 dpi, minor amounts of viral RNA were excreted by three out of ten chickens of this group (supplemental Table. 2). Virus excretion kineticsfor the HPAIV mono-infected chickens of group M6 (Fig. 1c, b vs. M6) showed a steady increase of virus shedding until death of the animals within 6 days. In co-infected groups, shedding of both LP-specific and HP-specific H7 RNA was evident, with the exception of group M1 where neither LP nor HP shedding was detected; with increasing amounts of HPAIV in the inoculation mixture, less LP H7-specific RNA was excreted (supplemental Table. 2).

In groups C1 and C3 no HP shedding was observed (C1 not shown in Fig. 1c, supplemental Table 2). Groups C5 and C5.7 did not shed statistically significant lower amounts of HPAIV RNA as compared to the HPAIV mono-infected groups M5 and M5.7 (Fig. 1c, supplemental Table 3b). Group C4 showed a singular HP excretion pattern with HPAIV RNA shedding receding to undetectable levels after a peak at 6 dpi; obviously co-infected chickens in this group cleared the HPAIV infection within the observation period. This is corroborated by the clinical picture where 6/8 inoculated birds survived.

#### HP H7N7 virus transmission from LP-co-infected donors to sentinel chickens requires higher HP inoculum doses

Virus transmission kinetics were assessed by co-housing four sentinel chickens with each of the groups at 1 dpi. Two sentinels each were sacrificed for immunohistochemistry (IHC) at 2 dpi. Effective transmissionwas evaluated by the development of morbidity/mortality (Fig. 1a, b dashed lines), presence of AIV RNA in OP and CL swab samples (Fig. 1d), and by seroconversion (supplemental Fig. 1) of the two remaining sentinels.

All contact chickens of HPAIV groups M and C, except those of groups C1-C4 and M1, excreted HPAIV (Fig. 1d and supplemental Table. 2), developed clinical signs and succumbed to the infection (Fig. 1a, b). Transmission of LPAIV as judged by virus excretion of sentinels was evidentby LP H7 pathotype-specific RT-qPCR in groups B, and C1-C5. No LPAIV excretion could be demonstrated in sentinels of group C5.7 (Fig. 1d, supplemental Table 2).

In summary, highly significant differences between the control groups B and M6 in survival and clinical score of sentinel animals were observed (each *p* < 0.001) (Fig. 1a, b). Furthermore, groups C3 vs. M3 and C4 vs. M4 showed a significant difference in survival (*p* < 0.001), while clinical scores did not differ significantly (*p* > 0.05). No statistical differences were observed in the remaining groupsC1 vs. M1, C5 vs.M5, and C5.7 vs. M5.7.

Results of the AIV NP antibody-specific seroconversion are shown in supplemental Fig. 3. All chickens found to be infected by molecular means and surviving until the end of the observation period (13 dpi) seroconverted.

#### Lack of macroscopic and histopathological findings in LP H7N7 infected chickens but characteristic lesions in HP H7N7 inoculated chickens at day 2 post infection

Necropsy of four chickens of each group (two inoculated and two sentinel birds) sacrificed at 2 dpi revealed no conspicuous pathological signs for AIV infection in either inoculated or contact chickens. This includes birds of all HPAIV mono-infected groups. Chickens inoculated with LPAIV and sacrificed at 2 dpi revealed no viral antigen-positive cells and no obvious histopathological alterations (supplemental Fig. 3).

**Fig. 2:**
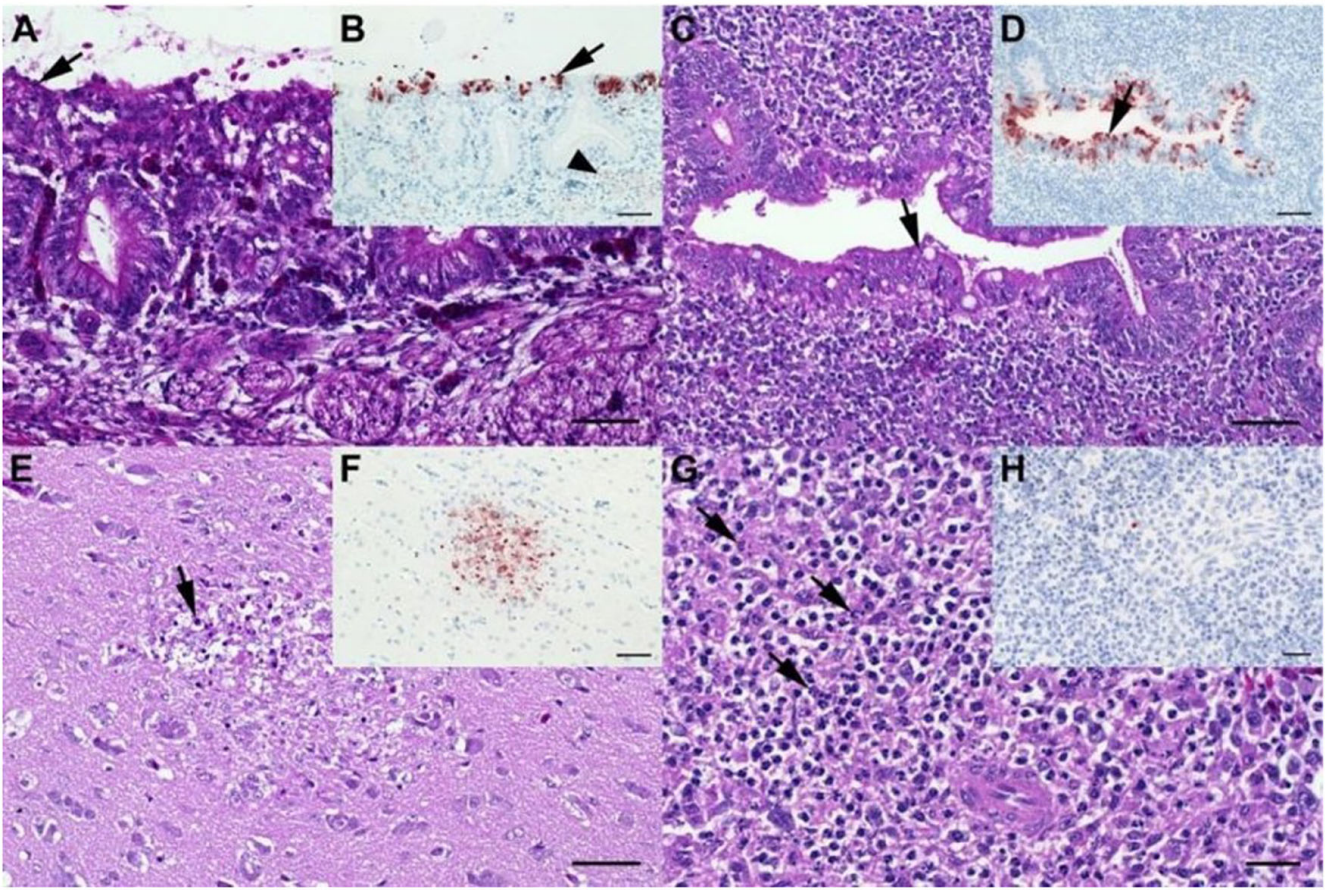
Histopathological findings in chickens (scheduled euthanasia at dpi 2) characteristic for HPAI. **a** Chicken, P17–882, group C5.7, respiratory mucosa. Moderate, multifocal, acute, necrotizing rhinitis with epithelial degeneration characterized by attenuation, loss of cilia (arrow), and sloughing. **b** Chicken P17–882, group C5.7, respiratory mucosa with coalescing foci of influenza A matrixprotein-immunoreactive (“antigen-positive”) morphologically intact and degenerated epithelial cells (arrow). Notably, there are scant immunoreactive granules in some of the submucosal nerves (arrowhead), suggestive of axonal spread. **c** Chicken, P17–903, group M5.7, cecum. Mild, oligofocal, acute, crypt epithelial degeneration with nuclear pyknosis (arrow), necrosis and sloughing. **d** Chicken, P17–903, group M5.7, cecum. Antigen-positive morphologically intact and degenerated crypt epithelia (arrow). **e** Chicken, P17–903, group M5.7, brain. Mild, oligofocal, acute, necrotizing polioencephalitis characterized by neuroglial cytoplasmic hypereosinophilia, nuclear pyknosis, karyorrhexis and loss (arrow), as well as an associated *status spongiosus* interpreted as inflammatory edema. **f** Chicken, P17–903, group M5.7, brain. Antigen-positive neuroglial cells within necrotizing lesion. **g** Chicken, P17–903, group M5.7, spleen. The periarteriolar lymphoid sheaths and follicles display moderate, coalescing apoptotic lymphocytes characterized by cytoplasmic hypereosinophilic shrinkage and nuclear karyorrhexis (arrows). **h** Chicken, P17–903, group M5.7, spleen. Rare, individual antigen-positive round cells can be detected in the white and red pulp. **a**, **c**, **e**, **g**: Hematoxylin eosin. **b**, **d**, **f**, **h**: Influenza A virus-matrixproteinIHC. **a**–**f**: bar = 50 µm. **g**, **h**: bar = 20 µm

In HPAIV infected chickens, in contrast, the most abundant histopathological finding at 2 dpi was mild to moderate, focal to multifocal, acute necrotizing rhinitis with epithelial degeneration and necrosis (groups M5-M6 and C4-C5.7; Fig. 2a; supplemental Fig. 2). Less frequently present was mild oligofocal, acute degeneration and necrosis of individual caecal crypt epithelia (Fig. 2c; supplemental Fig. 2) and mild oligo-focal to multifocal, acute, necrotizing polioencephalitis (Fig. 2e; supplemental Fig. 2a), respectively. Further mild to moderate lesions in other tissues are shown in Fig. 2g; supplemental Fig. 2b and 2d.

These results were corroborated by IHC revealing most abundant, oligofocal to coalescing influenza A matrixprotein-positive epithelial cells within the nasal cavities (Fig. 2b; supplemental Fig. 2c), caecal crypt epithelia (Fig. 2d; supplemental Fig. 2c), and the brains of chickens with polioencephalitis, respectively (Fig. 2f; supplemental Fig. 2c). Oligofocal, immunoreactive round cells interpreted as macrophages were present within the spleens of one to two chickens of groups M5.7, M6, and C5.7 (Fig. 2h; supplemental Fig. 2c). Other organs with scant focal immunoreactive parenchyma in individual chickens of groups M5-M6 were heart, lungs, and liver (supplemental Fig. 3C).

### In ovo experiment

ECEs at both 10 and 14 days of age were inoculated with either H7N7 LP AR915 or HP H7N7 AR1385 or mixtures thereof in 0.2 mL per egg as shown in Table 1b.

### LP H7N7 interferes with HP infection of embryonatedchicken eggs

**Fig. 3:**
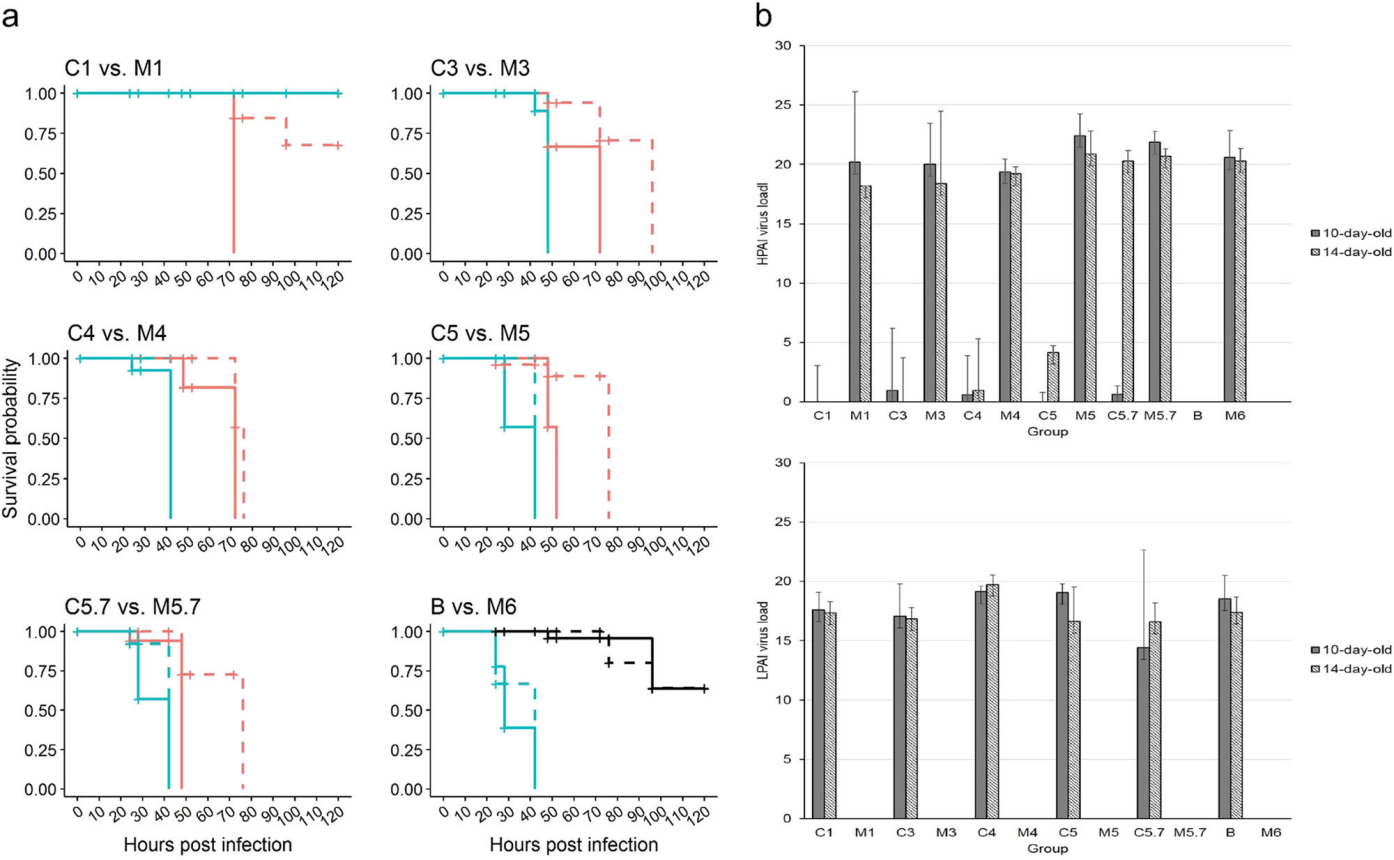
Results of the in ovo experiment. **a**. Survival probability of embryonated chicken eggs (ECEs) inoculated at 10 or 14 days of incubation with LP (group B, black lines in lower right panel “B vs. M6”) or HPAIV H7N7 (groups M1-M6, turquoise lines) or with LP/HP mixtures according to Table 1b (groups C1-C5.7, orange lines). Solid lines represent ECEs 10-day old and dashed lines 14-day old at inoculation. **b**. HP (upper panel) and LPAI (lower panel) H7N7 virus loads in amnio-allantoic fluids of ECEs aged 10-and 14-days at inoculation determined by H7 pathotype-specific RT-qPCRs (mean values representing “virus load” were calculated by substracting the measured Cq value from the threshold of detection at Cq = 38; individual values are presented in supplemental Table 3a)

Mean death time (MDT) values within an observation period of 4 days and viral load in amnio-allantoic fluids (AAFs) were used to compare mono- and co-infection groups. Significantly increased mortality and shortened MDT in groups M6 versus B, representing mono-infections with HP AR1385 and LP AR915 (inoculum each of 10^6^ EID_50_), was evident (*p* < 0.0001; quantitative measure: survival probability, Fig. 3a, B vs. M6, supplemental Table 4a,b). No significant (*p* > 0.5) difference was observed when comparing 10-day old and 14-day old eggs for each of these groups. Examination of virus loads in AAFs by generic AIV-M and pathotype-specific H7 LP- and H7 HP RT-qPCRs revealed pure LP and HP infections in the mono-infected groups B and M6, respectively (Fig. 3b, supplemental Table 5).

Independent of the virus dose at inoculation, the MDT of HP mono-infected ECEs was always significantly shorter, i.e., they died earlier, compared to LP/HP co-infected eggs (Fig. 3a, supplemental Table 4b). In addition, HP virus yield in all co-infected groups was strikingly lower compared to mono-infectionsbut increased with increasing HP inoculum titers,whereas no significant differences (*p* > 0.05) in LP virus load of co-infected groups was evident; interference of HP virus by LP co-infection was particularly dominant in 10-day old ECEs (Fig. 3b, supplemental Table 5). The only exceptions are groups C1 and M1 where no embryos died in M1, although all M1 eggs became infected (Fig. 3a, supplemental Table 5). In contrast, the death of all 5 (10-day old ECEs) and 3 of 5 ECEs (14-day old) in C1 must have been attributable mainly to LP replication as confirmed by pathotype-specific RT-qPCR (Fig. 3b, supplemental Table 5). ECEs inoculated at 10 days of incubation had a significantly shorter MDT compared to 14-day old ones in the co-infected but not in the mono-infected groups (Fig. 3a, supplemental Table 4b). However, this correlation was blurred by HPAIV mono-infections, and HP virus yield in AAFs did not vary with the inoculum dose and age of the ECE (Fig. 3b, supplemental Table 5).

#### Endotheliotropic-vascular systemic virus spread is characteristic for HP H7N7 in ECEs

**Fig. 4:**
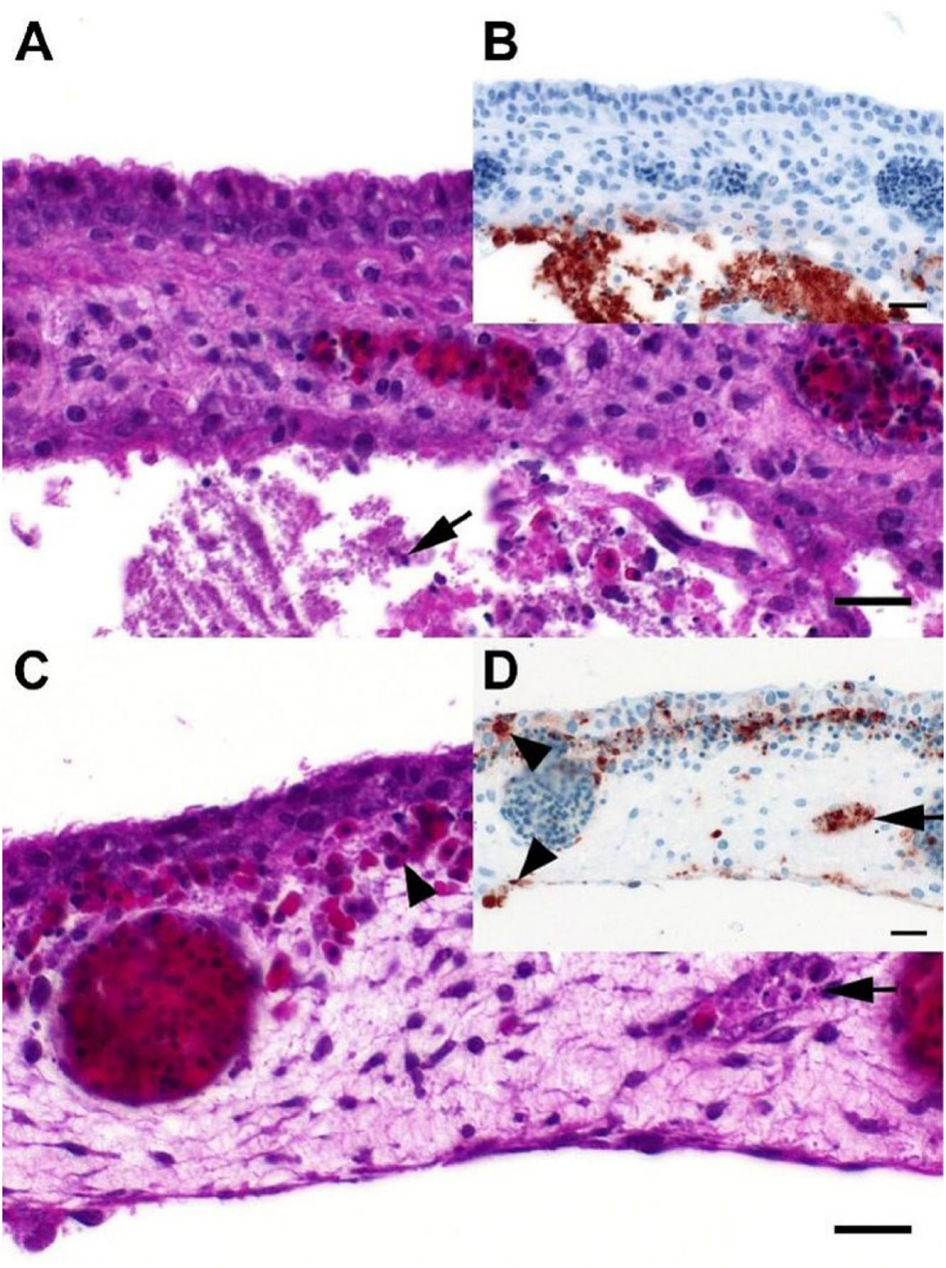
Histopathological findings in embryonated chicken eggs characteristic for LP and HP H7N7influenza A viruses exhibitingdistinct tropism in the chorioallantoic membrane. **a** Chicken, 14-day old embryo, P18–103, group B, 4 dpi, chorioallantoic membrane. There is necrosis and loss of the allantoic membrane and accumulation of cellular debris (arrow) and fibrin in the allantoic sac. **b** Chicken, 14-day old embryo, P18–103, group B, 4dpi, chorioallantoic membrane. Influenza A matrixprotein-immunoreactive (“antigen-positive”) cells and cellular debris accumulations restricted to the allantoic side of the chorioallantoic membrane. **c** Chicken, 12-day old embryo, P18–46, group C5.7, 4dpi, chorioallantoic membrane. Moderate, multifocal, acute, necrotizing vasculitis (arrow) with edema and extravascular accumulation of erythrocytes (arrowhead) interpreted as hemorrhage. **d** Chicken, 12-day old embryo, P18–46, group C5.7, 4dpi, chorioallantoic membrane. Influenza A-immunoreactivity is abundant in vascular endothelia (arrow) and blood-borne leukocytes and to a lesser degree epithelial cells (arrowheads) of both the chorionand the allantois (bottom). **a**, **c**: Hematoxylin eosin. **b**, **d**: Influenza A virus-matrixprotein IHC. **a**–**d**: bar = 20 µm

The trilamellar structure of the chorioallantoic membrane (CAM; inner layer = allantoic epithelium, intermediate mesenchymal layer and outer layer = chorionic epithelium) was clearly discernable in all investigated eggs/embryos^26^. Overall distribution patterns of virus antigen in the CAM and embryonal organs displayed a comparable pattern in 10-day old and 14-day old eggs (Fig. 4), and were attributable to the viral pathotype: Infection with LPAIV (embryos of group B) was mostly confined to the allantoic epithelium of the CAM (Fig. 4). In contrast, infection with HPAIV affected both the allantoic and chorionic epithelial layers, as well as endothelial cells within blood vessels of the mesenchymal layer of the CAM (Fig. 4, supplemental Figs. 4C and 4E). Furthermore, endotheliotropic systemic virus dissemination via the vascular system in embryos co-infected with HPAIV (groups C1-C5.7) was prominent with viral antigen present in endothelial and parenchymal cells of various internal organs (CNS, lung, liver, gizzard, and various parts of the gastrointestinal tract, and in some cases in the kidneys) (supplemental Fig. 3, supplemental Fig. 4C-F).

Examination of embryonic parenchymal organs (brain, liver, heart) by H7 pathotype-specific RT-qPCR revealed H7 LP RNA in a minority of tissue samples, mainly of 10-day old ECEs of some co-infection groups (C1-C5.7; supplemental Table 6a, red-colored values). Contamination from AAF during preparation cannot be fully excluded although no LP H7 RNA was detected in embryonic tissues of group B (LP H7 mono-infection). Presence of HPAIV RNA wasconfirmed in heart, brain and, liver tissues of both, 10-day old and 14-day old ECEs (supplemental Table 6a) but not in embryos of group B.

## Discussion

A shift of the susceptibility of the HACS from trypsin- to furin-like endoproteases essentially governs the emergence of an HP variant from its LP precursor^13,19,20,22,27,28^. Insertion of multiple basic amino acids^29–31^ but also intersegmental recombination and insertion of amino acids that fostered a bulging of the HACS^25,32,33^ mediated this switch in pathogenicity. Factors remained unknown that govern the likelihood of such spontaneous LP-to-HP mutations and skew the initial amplification and spread of the HP variant to ensure primacy over its LP precursor in the index bird. As we deeply studied the mutations of the applied LP/HPAIV matching pair in a former study, none of these mutations have been assigned to any specific functions, although the mutation N92D in NS-1 affected a position that has been associated with interferon escape^19^. We therefore performed co-infection experiments of LP and HP H7 viruses in chickens in vivo and in ovo to mimic the initial moments of amplification and spread following the de novo generation of HP variants. Subsequently, the HP variant must be transmitted within a chicken population, where a concurrent infection of the antigenically identical LP precursor is likely on-going as was the case in the layer flock from which our HP H7 variant originated^19^.

Our results demonstrated interference between LP and HP viruses that turned out to be highly unfavorable for the HP phenotype to gain primacy at LP/HP ratios exceeding 100:1. The factors responsible for interference have not been elucidated here but it seems likely to suspect that innate immune mechanisms such as interferon activity are involved^34^.We speculate that a switch to the HP phenotype has to occur very early after LPAIV infection to avoid interference by high loads of replicating LPAIV. In these cases, as mimicked by our co-infected groups C5 and C5.7, the HP variant prevailed, and substantial amounts of HP sufficient to infect sentinel animals were excreted. In contrast, in other co-infected groups receiving an inoculum with an unfavorable LP/HP ratio, such as C3 and C4, hampered HP virus excretion resulted, and transmission to sentinels failed. In these cases, HP mutants seemed to be unable to spread from the index animalwhile LP virus was excreted via oropharyngeal secretions and feces (groups C3 and C4). Escape of HP would depend on the death of the bird and exposure of susceptible hosts to systemically infected tissues. Such can be envisioned, e.g., by picking on decomposing carcasses^35^, contact with infected feather cones^36–38^, eggs^39,40^ or on mechanical transmission by hematophagous ectoparasites such as *Dermanyssus spp*^41^.

At the population level, transmission of HP mutants after escape from index birds depends on the presence of susceptible hosts. As former studies already showed, LPAIV precursor-specific immunity effectively reduced susceptibility to homologous HP infection and grossly decreasedHP transmission efficacy^42–44^ resembling immunization with a perfectly matching modified live virus vaccine. At flock level, the emerging HP variant would benefit from (i) a low prevalence of LP-specific adaptive immunity to avoid specific immune interference, and (ii) a low incidence of active LP precursor virus infection to prevent direct competition. Populations showing high seroconversion rates should be unlikely to propagate HP and may pose a low risk from an animal-disease-control perspective.

The in ovo experiment mirrored the inhibitory effects of an LP infection on the replication kinetics of co-inoculated HPAIV. We decided to compare 10-day old and 14-day old embryos due to developmental features in the embryo (e.g., new physical and functional barriers like the trilamellar structure of the CAM, access to tryptic proteases, etc.), that in turn may modify the distribution of AIV in embryonic tissues^45,46^. Characteristic differences regarding sites of replication of LP and HPAIVs in ECEs^47–50^ were confirmed for the current natural LP/HP pair of subtype H7N7. In particular, IHC analyses of the trilamellar CAM allowed an easy and clear-cut distinction between LP (infection of allantoic epithelium only) and HPAIV (cells of all three lamellae affected with characteristic endotheliotropism and vasculitis). Rarely, in embryos of group B (mono-infected by LP), immune-labeled cells were also found in the surface epithelia of the skin, nasal cavity, respiratory, and upper alimentary upper alimentary tract including the stomach; this pattern is suggestive for a canalicular spread of virus provided it had reached the amniotic cavity. Although inoculation was targeted to the allantoic sac, accidental lesion of the amnion cannot be excluded^50,51^. We confirmed previous results suggesting an increased resistance of 14-day old versus 10-day old ECEs to AIV infection^29,52,53^.

Considering all impediments that hamper HP variants to gain primacy over its LP precursor, the de novo emergence of HP viruses is likely a very rare event. Nevertheless, several de novo HP outbreakshave been detected during the last decade^8,17–19^. It is challenging to speculate that LP-to-HP mutation events in vivo actually might occur more frequently in LP-infected poultry flocksthan anticipated. Often, however, such conversion events might escape detection because HP virus remained trapped in the index birds or failed to spread in the population. Experimental LP-to-HP conversion of H7 virusesby serial passaging of LPAIVs has been successful^24,54^. Although HP phenotypes were generated eventually in several^29,55,56^ but not all attempts, a high number of passages was usually required. Yet, the true conversion rate of LP-to-HP may be underestimated.

The presented clinical, pathological and virological data, obtained by in ovo and in vivo co-infection experiments using a natural pair of LP and HP H7N7 viruses, revealed an intricate interference between the two phenotypes. HP variants generated by de novo mutation need to overcome a series of obstacles both in the index bird and in the index population to gain primacy. The in ovo model showed potential to determine, by IHC, tissue tropism and pathogenicity of AI viruses. Exploring different routes of inoculation (e.g., allantoic versus amniotic versus vascular) in 14-day old ECEs and subsequent deep sequencing of selected embryo tissues may also be appropriate to select pathogenicity variantsgenerated de novo or propagated from a minority population in the quasispecies of an isolate.

## Materials and methods

### Ethics statement

All experiments were carried out in biosafety level-3 (BSL-3) laboratory and animal facilities at the Friedrich-Loeffler-Institute (FLI, Germany) with permission of the FLI biorisk committee in accordance with a protocol legally approved by the Ethics Commission of the Ministry of Agriculture and the Environment of the State of Mecklenburg-Vorpommern, Germany (LALLF MV 7221.3–1.1–039/17).

### Virus origin and propagation

Viruses originated from two epidemiologically linked outbreaks in chicken layer farms in Germany in summer 2015. The two reference viruses constitute a natural pair of an LP precursor (A/chicken/Germany/AR915/2015 H7N7, AR915) and its HP descendant (A/chicken/Germany/AR1385/2015 H7N7, AR1385), differing, outside the HACS, by only very few mutations^19^. Infectivity titers are expressed as mean embryo infectious doses (EID_50_/mL) using isolates at passage level 2 in ECEs.

### Experimental design

#### In vivo co-infection experiment

In total, 168 white leghorn chickens, hatched from SPF ECEs (Lohmann Animal Health, Cuxhaven, Germany), were randomly assigned to 12 groups of 14 birds each at 6 weeks of age. Groups were housed in separate animal rooms. Each group tested AI-negative by serological and virological means (see below). Six groups (group M1-M6) were used to titrate clinical and pathohistological effects of the H7N7 HPAIV isolate AR1385 at doses of 10^1^ (group M1), 10^3^ (group M3), 10^4^ (group M4), 10^5^ (group M5), 10^5.7^ (group M5.7), or 10^6^ (group M6) EID_50_ in 0.5 mL inoculum per bird. Another five groups (group C1-C5.7) received mixtures of a constant dose of H7N7 LP (10^6^ EID_50_) and HP viruses at different concentrations as shown in Table 1a. Finally, one group (B) received 10^6^ EID_50_ in 0.5 mL per bird of the LPAIV H7N7 AR915 as a mono-infection. Oculo-oronasal inoculation mimicked a natural infection route in 10 chickens per group; four further chickens served as sentinels and were associated on dpi 1.

**Table 1a:** EID_50_/0.5 mL per chicken used for the in vivo experiment

Group	EID_50_/0.5 mL					
	10^1^	10^3^	10^4^	10^5^	10^5.7^	10^6^
C (+10^6^ LP)	C1	C3	C4	C5	C5.7	–
M	M1	M3	M4	M5	M5.7	M6
B	–	–	–	–	–	B

Experimental design (classification of the groups), 1a in vivo, 1b in ovo experiment. EID_50_ values refer to the HPAIV infection doses (groups C and M) and the LPAIV mono-infection dose (group B), respectively

#### Clinical score and survival rate

During the study period of 13 days, chickens were monitored and scored threetimes a day for clinical signs: 0 (normal/healthy), 1 (sick), 2 (severely sick), or 3 (dead). The highest score obtained at each day for a bird was used for statistical comparisons. Sick chickens showed one of the following symptoms: mild depression/tiredness and ruffled feathers, mild respiratory manifestations, facial edema, tentative feed intake or mild neurological signs. Severely sick birds showed two or more signs as described above and, in addition, cyanosis of the comb and the wattles, diarrhea, severe neurological signs (such as paralysis or convulsions). Moribund chickens reaching humane termination criteria were permanently drowsy and recumbent, could not be urged to move or showed severe dyspneic movement of the sternum. Such birds were humanely killed and registered as “3” (=dead) the day after. Morbidity and mortality indices were calculated according to the O.I.E. regulations for the Intravenous Pathogenicity Index (IVPI)^10^.

At dpi 2, four chickens per group (each two inoculated and two sentinel chickens) were sacrificed in deep isoflurane anesthesia and subjected to postmortem examination. These animals were not considered for mortality index calculations. At dpi 13, surviving chickens were finally bled.

### Sampling strategy

Serum samples were taken before virus exposure (dpi 0) and at 2 (birds sacrificed for histopathology), 6, and 13 dpi. Oropharyngeal (OP) and cloacal (CL) swabs to be collected from all virus-inoculated as well as all sentinel chickens were scheduled for dpi 0, 1, 2, 4, 6, 9, and 13 to assess virus shedding via the respiratory and digestive tracts. Birds were also swabbed when found dead or when they met the termination criteria. OP and CL swab samples were collected in 1 mL of serum-free cell culture medium and kept cooled at 4 °C until processed within 2 h after collection. Remaining swab supernatant was kept frozen at −70 °C.

### In ovo experiment

A similar co-infection experiment of LP AR915 and HP AR1385 H7N7 viruses was carried out in ovo using two series of 10-day old and 14-day old SPF ECEs. Infection of five eggs per group was done by the allantoic route according to standard protocols^10,47,57^. Inoculation doses of mono-infected and co-infected ECEs (groups M1–5.7 and C1–5.7 and B) are shown in Table 1b. Eggs were incubated at 37 °C and candled daily for embryonic vitality. After embryonic death or a maximum of 96 h of incubation eggs were chilled at 4 °C for a minimum of 4 h. Amnio-allantoic fluids (AAFs)  were harvested and assayed for HA as described^10^. Only eggs that were hemagglutination-positive in the AAF were considered for mean-death-time (MDT) calculations. Eggs with no HA titer (i.e., “no AIV-related death”) or which did not die within 4 days scored a value of 120 h post inoculation (hpi). In addition, RNA extracted from AAF was tested by RT-qPCR as described below.

**Table 1b:** EID_50_/0.2 mL per egg used for the in ovo experiment

Group	EID_50_/0.2 mL					
	10^1^	10^3^	10^4^	10^5^	10^5.7^	10^6^
C (+10^6^ LP)	C1	C3	C4	C5	C5.7	–
M	M1	M3	M4	M5	M5.7	M6
B	–	–	–	–	–	B

C = co-infection, M = mono-infection, B = mono LPAIV-infection

Furthermore, the embryo and the allantoic epithelium of two of the five eggs was dissected following the techniques described by Seekings^58^ and immersed in 10% neutral buffered formalin for histopathological analysis. Further embryonic tissues (brain, liver, and heart) of two eggs per group were sampled for RT-qPCR analysis. Virus present in AAF that was in contact with the embryo’s skin was removed by washing the embryos repeatedly and extensively in phosphate-buffered saline before incision for tissue removal.

### Detection of viral shedding and molecular pathotyping of AIV RNA

Viral RNA was extracted from swab fluids (chickens), AAF and tissue samples (ECE) using the NucleoMag®VET Kit (Macherey-Nagel GmbH & Co. KG, Düren, Germany) according to the manufacturer’s instructions and stored at −20 °C until use. By using quantitative real-time RT-PCR (RT-qPCR), presence of RNA of the influenza A virus matrix (M) gene was confirmed by the protocol of Hoffmann, et al.^59^. M-positive samples were further subtyped by H7 pathotype-specific RT-qPCRs, which allowed probe-assisted differentiation of the mono-basic and polybasic HACS of the LP and HP H7 pathotype^60^. All RT-qPCR reactions were performed in 25 μL volumes using the AgPath-ID RT-PCR kit (Ambion, Austin, TX, USA) and run on a CFX96 thermocycler machine (Bio-Rad).

### Serology

After heat inactivation for 120 min at 56 °C (safety precautions), all sera were examined for antibodies against the AIV nucleoprotein (NP) using an Influenza A Antibody Competition enzyme-linked-immunosorbent assay (ELISA) (ID Screen®, IDVET, Grabels, France) according to the manufacturer’s recommendations.

### Histopathology and immunohistochemistry

Selected tissues and three cross sections of the skull (nasal cavity and paranasal sinuses) from chickens sacrificed at 2 dpi as well as tissue samples from the in ovo experiments were fixed in 10% neutral buffered formalin,and processed for hematoxylin and eosin staining. The severity of necrotizing inflammation, epithelial degeneration and/or necrosis in the nasal mucosa, as well as lymphatic necrosis, apoptosis and/or depletion in the lymphatic organs was scored on an ordinal 4-step scale (0 = unchanged, 1 = mild, 2 = moderate, 3 = severe).

IHC was performed on serial sections to detect influenza A virus antigen using the avidin-biotin-peroxidase complex method (Vectastain PK 6100; Vector Laboratories, Burlingame, CA, USA) with citric buffer (10 mM, pH 6.0) pretreatment. Antigen detection was achieved with a monoclonal antibody (mAb) directed against an epitope of the influenza A matrixprotein (ATCC clone HB-64). 3-amino-9-ethylcarbazol served as the chromogen (Agilent Technologies, Santa Clara, CA, USA), and hematoxylin counterstaining. Validated positive and negative archival tissues, as well as replacement of the specific antibody by Tris-Buffered-Saline (TBS) served as controls. The distribution of parenchymal, as well as endothelial influenza A matrixprotein was evaluated on an ordinal 4-step scale (0 = none, 1 = focal/oligofocal, 2 = multifocal, 3 = coalescing/diffuse).

### Statistical analyses

The Mantel-Haenszel logrank test and the Mann Whitney test were used to compare survival rates and morbidity index as well as MDT values, respectively, applying the R software environment and the following packages: “stats”, “survival”, “survminer”, “gridExtra” and “ggplot2”. *P* values < 0.05 were considered significant. For comparisons between the total amount of virus shedding of HPAIV in the mono-infected and co-infected groups, area-under-the-curvegraphs were computed by using R software packages “stats”, “survival”, “survminer”, and “ggplot2”. The mean average of Cq values of all animals sampled at the indicated dpi in a specific group were calculated and used to draw the curves. Animals negative in RT-qPCR at that date scored with a value of 40.

## Electronic supplementary material

Supplemental tables and figures

read me file supplemental material
