# Supplementary material for: A viral race for primacy: co-infection of a natural pair of low and highly pathogenic H7N7 avian influenza viruses in chickens and embryonated chicken eggs
Source: Emerg Microbes Infect. 2018 Dec 5;7:204. doi: 10.1038/s41426-018-0204-0 (PMC6279742; doi:10.1038/s41426-018-0204-0)
Supplement: Supplementary file 1 — Supplemental tables and figures [file 41426_2018_204_MOESM1_ESM.docx]

**Supplemental material**

**Supplemental Table 1a:** p-values of clinical scores (supplement to Figure 1a).

| **Group** | **infected** | **sentinel** |
| --- | --- | --- |
| **C1 vs M1** | NA | 0.331753367 |
| **C3 vs. M3** | 5.58E-09 | 0.066627494 |
| **C4 vs. M4** | 2.35E-05 | 0.290733828 |
| **C5 vs. M5** | 0.8287837 | 0.333533006 |
| **C5.7 vs. M5.7** | 0.2372944 | 0.886651032 |
| **B vs. M6** | 2.78E-11 | 0.00038952 |

**Supplemental Table 1b:** p-values of survival probabilities (supplement to Figure 1b).

| **Group** | **infected** | **sentinel** |
| --- | --- | --- |
| **C1 vs M1** | 1 | 1 |
| **C3 vs. M3** | 4.16E-07 | 0.0000374 |
| **C4 vs. M4** | 2.08E-03 | 0.0000585 |
| **C5 vs. M5** | 0.000666797 | 0.1631753 |
| **C5.7 vs. M5.7** | 0.037529348 | 0.0101169 |
| **B vs. M6** | 3.22E-18 | 0.0000527 |

**Supplemental Table 2:** RT-qPCR results of *in vivo* experiments (supplement to Figures 1c-d); copies of viral genome equivalents are shown.

**Supplemental Table 3a:** Area-under-curve (AUC) values from viral shedding analyses of the inoculated chickens (supplement to Figure 1c).

|  |  | **AUC values** | |
| --- | --- | --- | --- |
| **Group** | **Shedding*** | **Co-infection group** | **Mono-infection group** |
| **C1 & M1** | OP | 0 | 0 |
| **C3 & M3** | OP | 0 | 35.206 |
| **C4 & M4** | OP | 46.7115 | 40.1555 |
| **C5 & M5** | OP | 36.48566667 | 48.637375 |
| **C5.7 & M5.7** | OP | 48.68225 | 58.0387619 |
| **B & M6** | OP | 0 | 53.929 |
| **C1 & M1** | CL | 0 | 0 |
| **C3 & M3** | CL | 0 | 46.4385 |
| **C4 & M4** | CL | 41.435 | 46.377 |
| **C5 & M5** | CL | 25.95425 | 43.797 |
| **C5.7 & M5.7** | CL | 35.434125 | 55.40552381 |
| **B & M6** | CL | 0 | 41.0825 |

*OP = oropharyngeal swab; *CL = cloacal swab

**Supplemental Table 3b:** P-values of viral shedding analyses (supplement to Figure 1c).

| **Group** | **CL*** | **OP*** |
| --- | --- | --- |
| **C3 vs. M3** | 0.02913886 | 0.06293646 |
| **C4 vs. M4** | 0.16270325 | 0.14744695 |
| **C5 vs. M5** | 0.58302491 | 0.79220779 |
| **C5.7 vs. M5.7** | 0.83066424 | 0.76190476 |
| **B vs. M6** | 0.00277843 | 0.00277843 |

*OP = oropharyngeal swab; *CL = cloacal swab

**Supplemental Table 4a:** Mean death time calculations of 10-and 14-day old embryonated chicken eggs (supplement to Figure 3a).

**Supplemental Table 4b:** P-values of *in ovo* experiments: (A) comparison of MDTs between co- and mono-infection groups in 10- and 14-day old embryonated chicken eggs (Mantel-Haenszel-logrank test) and (B) comparison of MDTs of each of the mono- and co-infection groups within 10- and 14-day old ECEs (supplement to Figure 3a).

(A)

|  | **ECEs** | |
| --- | --- | --- |
| **Group** | **10-day old** | **14-day old** |
| **C1 vs. M1** | 9.63E-07 | 0.023185644 |
| **C3 vs. M3** | 0.01332194 | 4.94E-05 |
| **C4 vs. M4** | 9.34E-06 | 3.41E-07 |
| **C5 vs. M5** | 1.60E-05 | 0.000315491 |
| **C5.7 vs. M5.7** | 0.00402611 | 9.34E-06 |
| **B vs. M6** | 1.60E-09 | 4.63E-09 |

(B)

| **Group** | **10- vs. 14-day old ECEs** |
| --- | --- |
| **C1** | 0.00134865 |
| **C3** | 0.010914202 |
| **C4** | 0.016791821 |
| **C5** | 0.014158931 |
| **C5.7** | 0.006742087 |
| **M1** | 1 |
| **M3** | 0.291840545 |
| **M4** | 0.28274546 |
| **M5** | 0.026888454 |
| **M5.7** | 0.235044451 |
| **M6** | 0.67087816 |
| **B** | 0.919851039 |

**Supplemental Table 5:** RT-qPCR results of harvested amnio-allantoic fluids of 10-and 14-day old embryonated chicken eggs infected with LP and/or HPAIV (*in ovo* experiment) (supplement to Figure 3b).

**Supplemental Table 6:** RT-qPCR results of tissues selected from of 10-and 14-day old embryonated chicken eggs infected with LP/HPAIV (*in ovo* experiment; co-infections).

**Supplemental Figure 1:**

Serologic reactions after infection with AIVs of subtypes H7N7 LP and/or HPAIV based on indirect NP-ELISA (OD650. IDEXX) on day 2, 6 and 13 pi. (A) shows co-infection groups C1-C5.7, (B) mono-infection groups M1-M5.7 and (C) control groups B (LP) and M6.
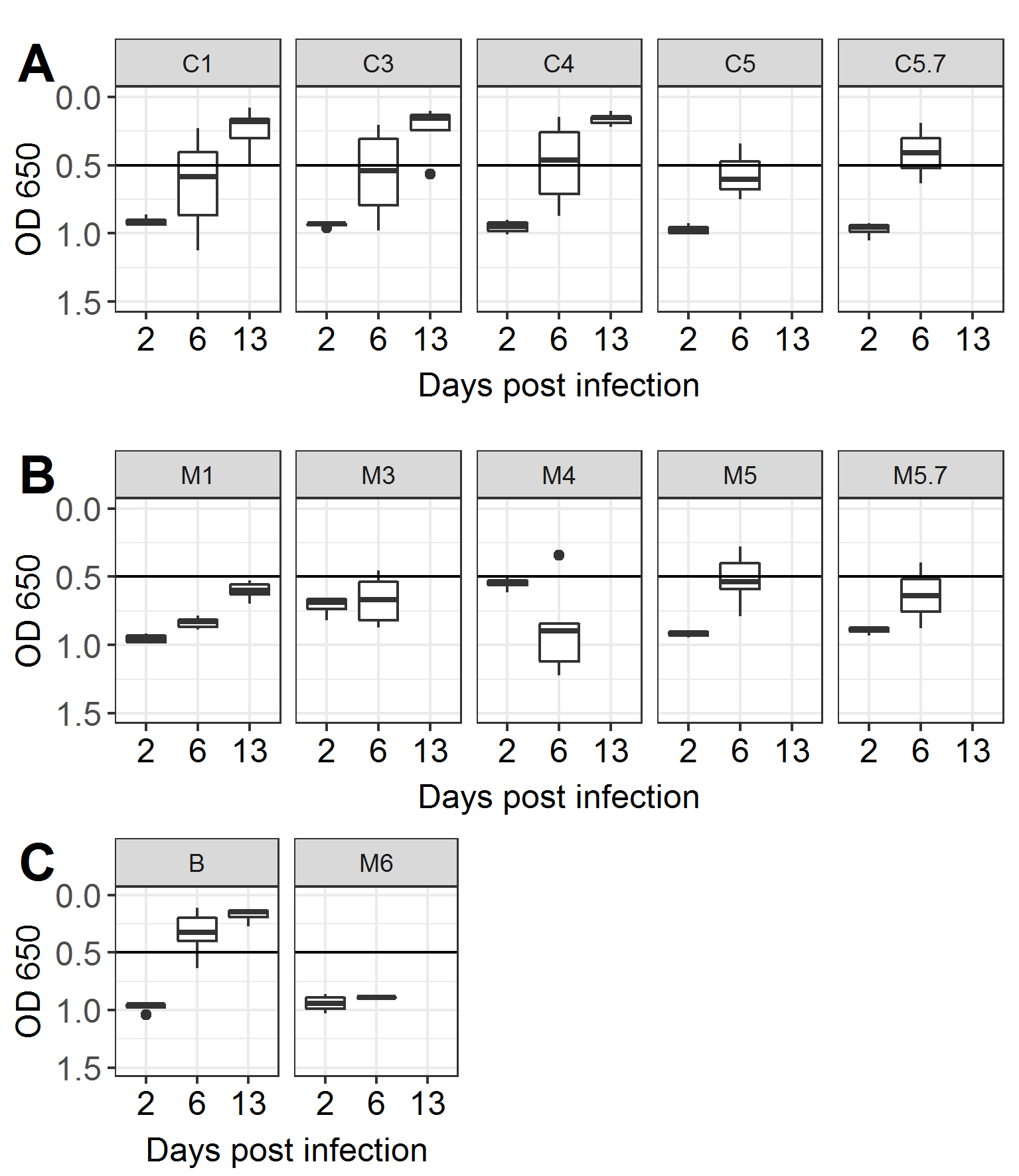


**Supplemental Figures 2a-d**: Histopathological findings and virus tropism as revealed by IHC in inoculated chickens sacrificed at 2 dpi.

(a) Severitiy of necrotizing inflammation. 0 = negative; 1 = mild; 2 = moderate; 3 = severe.

(b) Severity of lymphocytic apoptosis. 0 = negative; 1 = mild; 2 = moderate; 3 = severe.

(c) Distribution of parenchymal influenza A matrixprotein. 0 = negative; 1 = focal/oligofocal; 2 = multifocal; 3 = coalesing/diffuse.

(d) Distribution of endothelial influenza A matrixprotein. 0 = negative; 1 = focal/oligofocal; 2 = multifocal; 3 = coalesing/diffuse.

**Supplemental Figure 3:** Light microscopy revealed no obvious pathological findings in chicken infected with low pathogenic avian influenza (supplement to Figure 2).


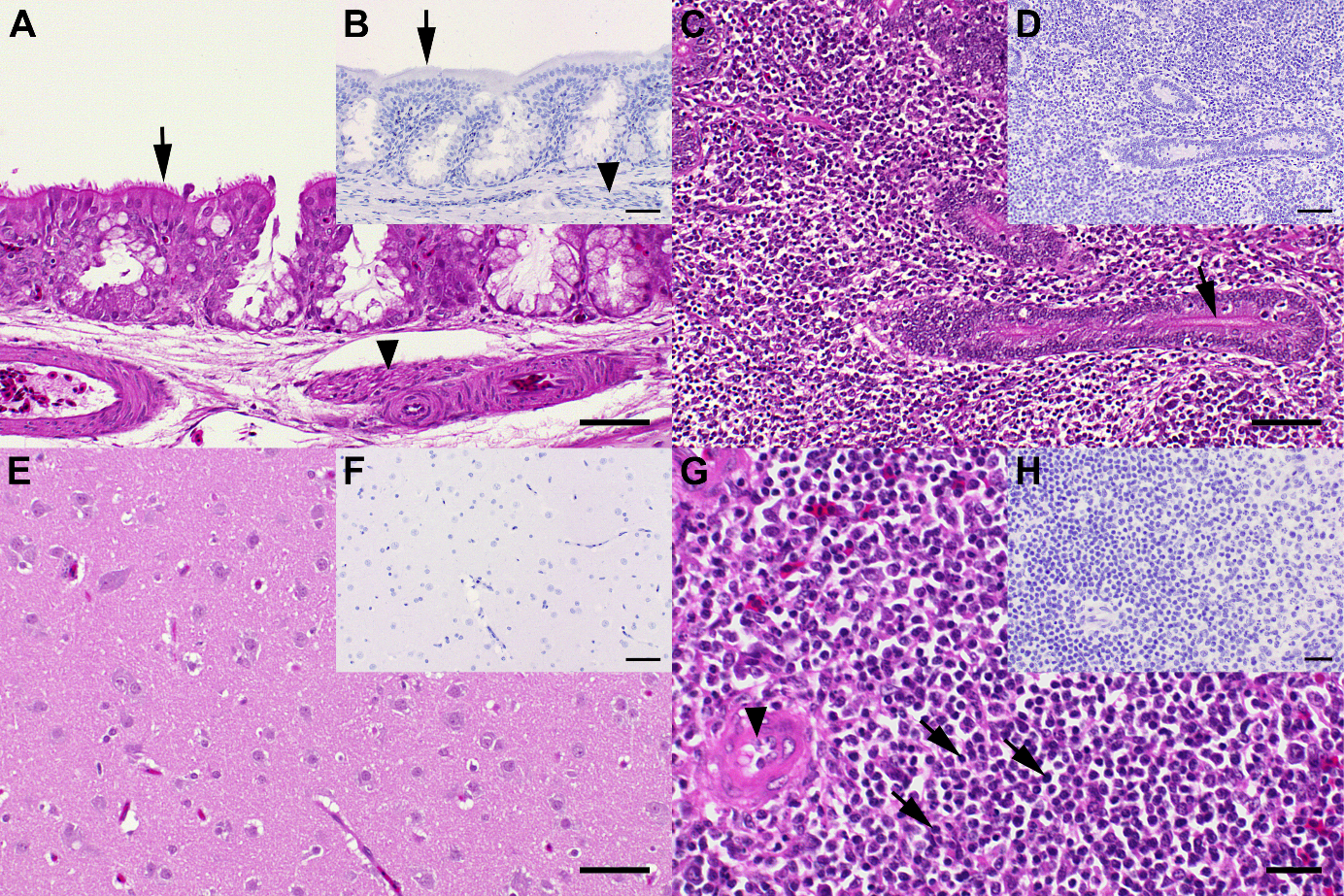


1. Chicken, P17-884, group B, scheduled euthanasia at 2 dpi, respiratory mucosa.

The respiratory mucosa is characterized by a pseudostratified columnar epithelium with prominent apical cilia (arrow) and multifocal intraepithelial mucous glands. The submucosa contains unremarkable blood vessels and nerves (arrowhead). **(B)** Chicken, P17-884, group B, scheduled euthanasia at 2 dpi, respiratory mucosa. The respiratory epithelial cells which can be identified based on their ciliated apical border (arrow) and the submucosal nerve fascicles (arrowhead) display no influenza A virus-matrixprotein immunoreactivity. **(C)** Chicken, P17-887, group B, scheduled euthanasia at 2 dpi, caecum. The caecal crypts extend deeply into the lymphoreticular tissue of the caecal tonsil and are lined by a columnar epithelium with typical brush border (arrow). **(D)** Chicken, P17-887, group B, scheduled euthanasia at 2 dpi, caecum. The lymphoreticular tissue of the caecal tonsils as well as the mucosa of the caecal crypts display no influenza A virus-matrixprotein immunoreactivity. **(E)** Chicken, P17-885, group B, scheduled euthanasia at 2 dpi, brain. Neurons and glia within the brain show no obvious pathological alterations. **(F)** Chicken, P17-885, group B, scheduled euthanasia at 2 dpi, brain. There is no influenza A virus-matrixprotein immunoreactivity within the brain. **(G)** Chicken. P17-887. group B scheduled euthanasia at 2 dpi, spleen. A small artery (arrowhead) is surrounded by small differentiated lymphocytes with round,, heterochromatic nuclei (arrows) forming the periarteriolar lymphoid sheath. **(H)** Chicken, P17-887, group B scheduled euthanasia at 2 dpi, spleen. There are no influenza A virus-matrixprotein immunoreactivity cells within the lymphoreticular tissue of the spleen. A. C. E. G: Hematoxylin eosin. B. D. F. H: Influenza A virus-matrixprotein IHC, avidin-biotin-peroxidase complex method, using as first antibody a murine monoclonal antibody directed against the matrixprotein of anti-influenza A virus (strain PR8 (A/PR/8/34. H1 N1); clone M2-1C6-4R3 (ATCC® HB-64™). American Type Culture Collection. Manassas. USA), 3-amino-9-ethyl-carbazol as chromogen and hematoxylin counterstain. A-F: bar = 50 µm. G. H: bar = 20 µm.

**Supplemental Figure 4a-f**: Histopathological findings and virus tropism in chorioallantoic membrane and embryonal organs (supplement to Figure 4).

(a) 10-day old chicken embryos; Severity of necrotizing inflammation. 0 = negative; 1 = mild; 2 = moderate; 3 = severe.

(b) 14-day old chicken embryos; Severity of necrotizing inflammation. 0 = negative; 1 = mild; 2 = moderate; 3 = severe.

(c) 10-day old chicken embryos; Distribution of parenchymal and epithelial influenza A matrixprotein. 0 = negative; 1 = focal/oligofocal; 2 = multifocal; 3 = coalesing/diffuse.

(d) 10-day old chicken embryos; Distribution of endothelial influenza A matrixprotein. 0 = negative; 1 = focal/oligofocal; 2 = multifocal; 3 = coalesing/diffuse.

(e) 14-day old chicken embryos; Distribution of parenchymal and epithelial influenza A matrixprotein. 0 = negative; 1 = focal/oligofocal; 2 = multifocal; 3 = coalesing/diffuse.

(f) 14-day-old chicken embryos; Distribution of endothelial influenza A matrixprotein. 0 = negative; 1 = focal/oligofocal; 2 = multifocal; 3 = coalesing/diffuse.

**Statistical analyses**

The Mantel-Haenszel logrank test and the Mann Whitney test were used to compare survival rates and morbidity index as well as MDT values, respectively, applying the R software environment and the following packages: “stats”, “survival”, “survminer”, “gridExtra” and “ggplot2”. P values <0.05 were considered significant. For comparisons between the total amount of virus shedding of HPAIV in the mono- and co-infected groups, area-under-the-curve graphs were computed by using R software packages “stats”, “survival”, “survminer” and “ggplot2”. The mean average of Cq values of all animals sampled at the indicated dpi in a specific group were calculated and used to draw the curves. Animals negative in RT-qPCR at that date scored with a value of 40.
