# Supplementary material for: A viral race for primacy: co-infection of a natural pair of low and highly pathogenic H7N7 avian influenza viruses in chickens and embryonated chicken eggs
Source: Emerg Microbes Infect. 2018 Dec 5;7:204. doi: 10.1038/s41426-018-0204-0 (PMC6279742; doi:10.1038/s41426-018-0204-0)
Supplement: Supplementary file 2 — read me file supplemental material [file 41426_2018_204_MOESM2_ESM.docx]

**README File for Supplementary Material**

The supplemental material file includes all supplemental figures and tables that are not included but referred to in the main manuscript as well as a description of statistical analyses.

**Packing list:**

**Supplemental Tables:**

- **Supplemental Table 1a**: p-values of clinical scores (supplement to Figure 1a).
- **Supplemental Table 1b**: p-values of survival probabilities (supplement to Figure 1b).
- **Supplemental Table 2**: RT-qPCR results of *in vivo* experiments (supplement to Figure 1c-d); copies of viral genome equivalents are shown.
- **Supplemental Table 3a**: Area-under-curve (AUC) values from viral shedding analyses of the inoculated chickens (supplement to Figure 1c).
- **Supplemental Table 3b**: P-values of viral shedding analyses (supplement to Figure 1c).
- **Supplemental Table 4a:** Mean death time calculations of 10-and 14-day old embryonated chicken eggs (supplement to Figure 3a).
- **Supplemental Table 4b:** P-values of *in ovo* experiments: (A) comparison of MDTs between co- and mono-infection groups in 10- and 14-day old embryonated chicken eggs (Mantel-Haenszel-logrank test) and (B) comparison of MDTs of each of the mono- and co-infection groups within 10- and 14-day old ECEs (supplement to Figure 3a).
- **Supplemental Table 5:** RT-qPCR results of harvested amnio-allantoic fluids of 10-and 14-day old embryonated chicken eggs infected with LP and/or HPAIV (*in ovo* experiment) (supplement to Figure 3b).
- **Supplemental Table 6:** RT-qPCR results of tissues selected from of 10-and 14-day old embryonated chicken eggs infected with LP/HPAIV (*in ovo* experiment; co-infections).

**Supplemental Figures:**

- **Supplemental Figure 1:** Serologic reactions after infection with AIVs of subtypes H7N7 LP and/or HPAIV based on indirect NP-ELISA (OD650. IDEXX) on day 2, 6 and 13 pi. (A) shows co-infection groups C1-C5.7, (B) mono-infection groups M1-M5.7 and (C) control groups B (LP) and M6.
- **Supplemental Figures 2a-d**: Histopathological findings and virus tropism as revealed by IHC in inoculated chickens sacrificed at 2 dpi.
  - (a) Severitiy of necrotizing inflammation. 0 = negative; 1 = mild; 2 = moderate; 3 = severe.
  - (b) Severity of lymphocytic apoptosis. 0 = negative; 1 = mild; 2 = moderate; 3 = severe.
  - (c) Distribution of parenchymal influenza A matrixprotein. 0 = negative; 1 = focal/oligofocal; 2 = multifocal; 3 = coalesing/diffuse.
  - (d) Distribution of endothelial influenza A matrixprotein. 0 = negative; 1 = focal/oligofocal; 2 = multifocal; 3 = coalesing/diffuse.
- **Supplemental Figure 3:** Light microscopy revealed no obvious pathological findings in chicken infected with low pathogenic avian influenza (supplement to Figure 2).
- **Supplemental Figure 4a-f**: Histopathological findings and virus tropism in chorioallantoic membrane and embryonal organs (supplement to Figure 4).
  - (a) 10-day old chicken embryos; Severity of necrotizing inflammation. 0 = negative; 1 = mild; 2 = moderate; 3 = severe.
  - (b) 14-day old chicken embryos; Severity of necrotizing inflammation. 0 = negative; 1 = mild; 2 = moderate; 3 = severe.
  - (c) 10-day old chicken embryos; Distribution of parenchymal and epithelial influenza A matrixprotein. 0 = negative; 1 = focal/oligofocal; 2 = multifocal; 3 = coalesing/diffuse.
  - (d) 10-day old chicken embryos; Distribution of endothelial influenza A matrixprotein. 0 = negative; 1 = focal/oligofocal; 2 = multifocal; 3 = coalesing/diffuse.
  - (e)14-day old chicken embryos; Distribution of parenchymal and epithelial influenza A matrixprotein. 0 = negative; 1 = focal/oligofocal; 2 = multifocal; 3 = coalesing/diffuse.
  - (f) 14-day-old chicken embryos; Distribution of endothelial influenza A matrixprotein. 0 = negative; 1 = focal/oligofocal; 2 = multifocal; 3 = coalesing/diffuse.

**Contact information:**

In case you have questions regarding the supplemental material, please feel free to contact us at [timm.harder@fli.de](mailto:timm.harder@fli.de), or [annika.graaf@fli.de](mailto:annika.graaf@fli.de)
